# Accuracy of Tri-ponderal Mass Index and Body Mass Index in Estimating Insulin Resistance, Hyperlipidemia, Impaired Liver Enzymes or Thyroid Hormone Function and Vitamin D Levels in Children and Adolescents

**DOI:** 10.4274/jcrpe.galenos.2019.2018.0279

**Published:** 2019-11-22

**Authors:** Neşe Akcan, Rüveyde Bundak

**Affiliations:** 1 Near East University Faculty of Medicine, Department of Pediatric Endocrinology, Nicosia, Cyprus; 2 Kyrenia University Faculty of Medicine, Department of Pediatric Endocrinology, Kyrenia, Cyprus

**Keywords:** Body mass index, hyperlipidemia, impaired liver enzymes, insulin resistance, tri-ponderal mass index

## Abstract

**Objective:**

Tri-ponderal mass index (TMI) has been reported to estimate body fat more accurately than body mass index (BMI). This study aimed to compare the efficacy of TMI and BMI in predicting insulin resistance (IR), hyperlipidemia, impaired liver enzymes or thyroid hormone function and vitamin D concentration.

**Methods:**

One hundred and forty-three overweight or obese children, based on BMI-standard deviation (SD) scoring (BMI-SDS) were studied retrospectively. TMI thresholds for overweight status were 16.0 kg/m3 for boys and 16.8 kg/m3 for girls and 18.8 kg/m3 for boys and 19.7 kg/m3 for girls for obese status.

**Results:**

Twenty-two overweight and eight obese children by BMI-SDS were classified as normal by TMI. Of the overweight children 22 (22.7%) had IR and IR was detected in 2 of 8 obese children with normal TMI. There was no increase in liver enzymes in any of the children with normal TMI. Forty-four obese children were overweight according to TMI and IR was detected in 40.9%. Thyroid stimulating hormone levels were significantly higher in BMI-based obese children. Vitamin D levels were similar in all groups of both classifications.

**Conclusion:**

When TMI was used there may be a risk of overlooking IR. However, if it is assumed that liver enzymes are elevated as a result of visceral adiposity, TMI can be used as an auxiliary parameter to show visceral effects of adiposity. Normal TMI may indicate that visceral organ functions have not deteriorated yet. More studies are needed to evaluate TMI as a clinical tool.

**What is already known on this topic?**

Recently, the tri-ponderal mass index (TMI) has been reported as an alternative to body mass index (BMI). TMI has been reported to be nearly stable throughout adolescence and that it may estimate body fat levels more accurately than BMI, especially in adolescents.

**What this study adds?**

This study documented the usability of the proposed TMI values in Turkish children and investigated the relationship between TMI and some biochemical parameters. To the best of our knowledge, this is the first study to investigate the power of TMI as a predicter of liver enzyme concentrations. This is also the first application of TMI in Turkish children.

## Introduction

Childhood obesity is a major health problem of worldwide concern (1,2,3). During the past 20 years, the proportion of obese children and adolescents has significantly increased in most countries (1,2,3). Obesity in adolescents is a major risk factor for adulthood obesity (3). Childhood obesity is also strongly linked to comorbidities such as hypertension, hyperlipidemia, impaired glucose metabolism and type 2 diabetes, obstructive sleep apnea, non-alcoholic fatty liver disease and metabolic syndrome in childhood or later in life (3). Body mass index (BMI) is commonly used to diagnose obesity in children and adolescents. Recently, the tri-ponderal mass index (TMI) has been reported to be more stable throughout childhood and adolescence and to estimate body fat levels more accurately than BMI, especially in adolescents which is supported by y dual-energy X-ray absorptiometry (4,5). In line with the increasing interest on TMI, recent studies of TMI in both obese (4,5,6,7,8,9) and non-obese children (10) have been published. The aim of this study was to compare the efficacy of BMI and the recently proposed TMI in forecasting insulin resistance (IR), hyperlipidemia, impaired liver enzymes, thyroid hormone function and vitamin D concentrations.

## Methods

### Participants

In this retrospective study, a medical chart review was performed to collect data from the Pediatric Endocrinology Outpatient Clinics of the Near East University, Nicosia, Northern Cyprus. The medical records of all children and adolescents seen between January 2016 and December 2017 with a diagnosis of obesity were investigated. Initial data selection sought children and adolescents aged six to18 years with obesity or overweight according to their BMI-standard deviation (SD) score (BMI-SDS) at their first visit to the endocrine clinic and followed at the endocrine clinic for at least one year. Children with a BMI-SDS between +1.0 and +2.0 were accepted as overweight and those with a BMI-SDS greater than or equal to +2.0 as obese. Patients excluded from the study included those with syndromatic obesity, endocrine disorders associated with obesity such as hypothyroidism, Cushing’s syndrome, hypothalamic obesity and postcranial surgery or with non-endocrine chronic illness which require medications that might impact body weight (systemic steroids, psychiatric medications) and patients with missing data.

### Clinical and Biochemical Parameters

Routine clinical follow-up of patients at every clinic visit (usually every 4-6 months) during the time period of the study, included measurement of weight (with underwear, using a standard Seca digital weight scale), height (using a commercial Harpenden-Holtain stadiometer) and evaluation of pubertal stage according to the criteria of Marshall and Tanner. BMI and TMI were calculated as weight in kilograms divided by height in meters squared (kg/m^2^) and as weight divided by height cubed (kg/m^3^), respectively. The SDS of height, weight, and BMI were calculated according to the data of Neyzi et al (11) for Turkish children and adolescents. Considering that TMI is more stable in children and adolescents (4,5), established TMI thresholds used in the study. TMI thresholds used in the study to diagnose overweight status were 16.0 kg/m^3^ for boys and 16.8 kg/m^3^ for girls and were 18.8 kg/m^3^ for boys and 19.7 kg/m^3^ for girls to diagnose obese status (4). Fasting blood glucose, insulin, homeostasis model assessment-IR (HOMA-IR), high density lipoprotein (HDL) and low density lipoprotein (LDL) cholesterol, triglycerides (TG), total cholesterol (TC), liver function enzymes, thyroid hormones and *25*-*hydroxyvitamin D [*25(OH)D_3_*] *were evaluated. HOMA-IR was used to evaluate IR using the formula: HOMA-IR=[insulin (mU/l) × glucose (mmol/l)]/22.5 (3). The HOMA-IR thresholds of Turkish children were used to define IR as follows: 2.22 for prepubertal girls; 2.67 for prepubertal boys; 3.82 pubertal girls; and 5.22 for pubertal boys (12). TC ≥200 mg/dL and TG ≥150 mg/dL (≥1.69 mmol/L) were accepted as high (13). Thresholds for liver enzymes were accepted according to laboratory references [serum glutamic oxaloacetic transaminase (SGOT): 5-34 U/L, serum glutamic-pyruvic transaminase (SGPT): SGPT: 0-55 U/L]. Instead of a sex-specific cut-off for HDL, a single cut-off was used which was <1.03 mmol/L or <40 mg/dL as proposed by the International Diabetes Federation consensus definition of metabolic syndrome in children and adolescents (13). Vitamin D status was classified as sufficiency (>50 nmol/l or >20 ng/mL), insufficiency (30-50 nmol/l or 12-20 ng/mL) and deficiency (<30 nmol/l or <12 ng/mL), based on the consensus statement of the Endocrine Society (14). Abdominal ultrasound (USG) was performed to detect non-alcoholic fatty liver disease in patients with IR and/or elevated liver enzymes.

### Statistical Analysis

The Statistical Package for Social Sciences Software (SPSS 21, Chicago, IL, USA) was used for data analysis. Skewness and Kurtosis Z-values and Shapiro-Wilk’s test were used to test the distribution of data. All dependent variables are not normally distributed for each category of an independent variable (Shapiro-Wilk’s test p<0.05 and Skewness and Kurtosis Z-values were not between -1.96 to +1.96). Thus, non-parametric methods was used in data analysis. The Kruskal-Wallis H test was used to determine if there were statistically significant differences between more than two groups of an independent variable on a continuous or ordinal dependent variable whereas Mann-Whitney U test was used to determine if there were statistically significant differences between two groups. Finally, a chi-square test was used for testing relationships between categorical variables from a single population. All continuous variables were expressed as median, maximum and minimum values. Statistical significance was assumed when p<0.05.

## Results

A total of 143 patients were enrolled in the study. Of the total cohort, 58% (n=83) were female and 42% (n=60) were male. The mean±SD age of the patients was 11.1±2.9 (range 6.3-17.6) years. Based on BMI-SDS, the overweight group consisted of 37 patients (25.9%) and the obese group of 106 patients (74.1%), respectively. When the study sample was classified based on TMI thresholds, three groups were identified. These were normal 21% (n=30), overweight 41.3% (n=59) and obese 37.8% (n=54). Twenty-two overweight and eight obese children were classified as normal by TMI. No patient classified as overweight by BMI-SDS was classified as obese by TMI. Forty-four obese children were classified as overweight according to TMI. There were 54 (37.7%) patients who were classified as obese, based on both BMI-SDS and TMI (Table 1).

The median values of fasting blood glucose, insulin, HOMA-IR, TC, HDL, LDL, TG, SGOT, SGPT, 25(OH)D_3_, thyroid stimulating hormone (TSH) and free thyroxine (fT4) are presented in Table 2. The median values of serum TG, SGOT and SGPT differed within the groups according to TMI classification. The serum levels of TG, SGOT and SGPT in patients with normal TMI were significantly lower than those of both obese and overweight patients (Table 2). Median values of fasting blood glucose and TSH concentratons significantly differed between the overweight and obese patients based on BMI-SDS classification (Table 2). TMI classification did not effect the median values of thyroid hormones. However, all patients with normal TMI had normal TSH values. According to the BMI classification, all patients with elevated TSH were obese (Table 3). Serum 25(OH)D_3_ levels were similar in all groups according to both classification (Table 2, 3).

The incidence of IR in the total study group was 37.1% according to HOMA-IR thresholds for Turkish children. Based on BMI-SDS, eight (21.6%) of the overweight patients and 45 (42.5%) of the obese patients had IR. Based on TMI, seven (23.3%) of the normal, 21 (35.5%) of the overweight, 25 (46.3%) of the obese patients had IR (Table 3). The frequency of IR was significantly higher in obese children than in overweight when BMI was used to classify the study group (Table 3). Moreover, when we classified the study group according to BMI, another parameter that was significantly different between the obese and overweight groups was the frequency of low HDL levels. However only the frequency of elevated SGOT differed, although not significantly, within the groups when classified according to TMI (p=0.054) (Table 3).

22.7% of overweight children with normal TMI had IR, 9.1% high TC, 50% had LDL >100 mg/dL and 4.5% had low HDL and high TG. Two of eight obese children (BMI-SDS> +2) with normal TMI had IR and low HDL. There was no increase in liver enzyme levels in any child with normal TMI (Table 4). Forty-four obese children, who were overweight according to TMI, had IR 40.9%, low HDL 34.1% and at least one elevated liver enzyme was present in 11.4%. Isolated IR was detected in 46.3% of 54 patients who were obese according to the both BMI-SDS and TMI (Table 4).

In all insulin resistant cases (n=53), hepatosteatosis was observed in 15 (28.3%) patients (n=6, 40% female; n=9, 60% male) and at least one elevated liver enzyme was detected in seven (13.2%) patients of whom seven also had elevated SGOT and five had elevated SGPT. None of these seven had normal TMI, whereas two of them had overweight TMI value and the remaining five were obese by TMI (Table 5). All seven had at least Grade 2 hepatosteatosis on abdominal USG. Conversely, only eight (17.4%) of the patients who had IR without an increase in liver enzymes (n=46) had hepatosteatosis on abdominal USG (Grade 1 hepatosteatosis n=6; Grade 2 hepatosteatosis n=2). None of these patients had Grade 3 stetatosis. Remarkably, none of these eight patient had normal TMI value (Table 5).

## Discussion

This study investigated the usability of the proposed TMI values in Turkish children. The relationships between TMI and some biochemical parameters were also presented. This study compares the utility of TMI and BMI in forecasting IR, hyperlipidemia, impaired liver enzymes or thyroid hormone functions and 25(OH)D_3_ level. The current study is the first in Turkish children and to the best of our knowledge is the first to investigate the use of TMI in predicting abnormalities in liver enzymes.

The use of BMI as a surrogate of adiposity is especially problematic in the pediatric population, because the relative contributions of fat mass and lean body mass to body weight vary by age, sex, pubertal status, and population ancestry. Annual increases in BMI from midchildhood onward are largely because of increases in lean body mass rather than to increases in fat mass and differences in BMI percentiles indicate differences in fat mass only for high percentiles of BMI (15). If the goal is to define overweight status in children and adolescents based on percentiles of body fat or visceral adiposity, BMI-SDS may be overdiagnosing adolescents as overweight (4). Thus, the debate on overdiagnosis of overweight using BMI has been highlighted recently (4,5,15,16). This overdiagnosis may increase health care-related costs and also cause stress in both families and patients (4). Thus, if we use TMI, the number of children who are diagnosed as overweight or obese is likely to decrease. This is important because adolescents may be more sensitive than adults to being classified as overweight (4). Indeed, in our study, 22 overweight and eight obese children were classified as normal when we used TMI and there were no patients that TMI classified as obese while BMI-SDS classified as overweight. However, when we try to prevent overdiagnosis of overweight with BMI, the patient at risk should not be overlooked. So, both approaches may have some risks and undesirable consequences.

Both blood glucose and TSH were significantly different by BMI classification compared to TMI classification. Although, mean values of insulin and HOMA-IR were not significantly different between the BMI overweight and BMI obese groups, the frequency of IR according to the cut off values based on age and puberty, pointed to significant differencee in these two groups. Higher fasting glucose levels in obese patients than in those who are overweight may be expected, consistent with an increasing frequency of IR. Thus BMI may reflect IR and associated higher glucose values better than TMI. In contrast, using TMI classification, mean values of serum TG, SGOT and SGPT in patients with normal TMI, were significantly lower than those of both obese and overweight patients classified by BMI. Moreover, in our study, none of the insulin resistant cases with elevated liver enzymes or none of the insulin resistant cases with USG-proven hepatosteatosis had normal TMI. No patient with a normal classification by TMI had elevated liver enzymes. Although BMI has been reported as a good predictor of elevated SGPT in adolescents (17,18), the accuracy of the TMI in detecting impaired liver enzymes seems to be better than BMI. It has been reported that TMI may estimate body fat percentage more accurately than BMI (4,5). Accordingly, all of the study findings that present the correlation between TMI and liver enzymes or hepatosteatosis can be considered to support this recent information of estimation body fat percentage. The gold standard dual-energy X-ray absorptiometry is not always practical for screening body fat percentages, especially in children, so a simple calculation of TMI may be a practical approach to the assessment of increase in body fat. Moreover, TMI offers certain cut off values and does not need age-specific percentiles like as BMI, and thus provides ease of use (4,5). This may be particularly helpful in identifying a child with a higher risk of visceral adiposity and may also be helpful in referring these at-risk patients to the pediatric endocrine clinic. Conversely, some criticism also exists regarding TMI usage (6,7). It has been reported that fat distribution is more important than body fat percentage in determining adult obesity-related outcomes, such as type 2 diabetes. TMI does not account for fat distribution without distinguishing fat mass from muscle mass (6). Moreover, in contrast to studies which support TMI, BMI-SDS, BMI and waist circumference have also been reported as the most relevant anthropometric markers to predict metabolic risk in youth and these markers have been reported as superior to TMI (7).

In our study, USG-proven hepatosteatosis was found to be slightly more predominant in males. Although the frequency of IR in our group of patients was 37.1%, at least one high liver enzyme was present in seven and USG-proven hepatosteatosis was detected only in 15 insulin resistant cases respectively. This means that laboratory-demonstrated IR may not always correlate with visceral adiposity which causes organ damage or dysfunction. Moreover, the frequency of IR was close to 50% in patients who were evaluated as obese in both classifications. From this, it can be assumed that there may be other factors contributing to the formation of both IR and visceral adiposity, other than weight gain versus height, calculated by either method. The reasons for this difference may reside in genetic or ethnic differences, to variable socioeconomic status, environment or and to interactions among these variables (4). However, this study highlighted that these multifactorial effects on IR may be more correlated with BMI whereas TMI may be more sensitive to detect multifactorial causes leading to visceral adiposity.

The current study also tried to demostrate the relations between serum lipid levels and weight versus height by both BMI and TMI. Only the frequency of low HDL levels was shown to be correlated with BMI, while the mean TG concentrations were significantly different when using the TMI classification. However, many studies report an association of BMI and lipid levels in children (19,20,21,22). Although a statistically significant association between LDL concentration and BMI has been determined in a population-based, cross-sectional study (19) or BMI has been reported to correctly identify 77% of the total dyslipidemic disorders in obese children (22), our study did not show any relation between both BMI or TMI and LDL concentrations or total dyslipidemic disorders. However, the initial categorization of overweight or obesity was BMI based. It would be of interest to increase the sample size and the include children with normal BMI in future studies of BMI and TMI and lipid abnormalities in young people.

In addition, this study is the first study to test the relation between vitamin D concentrations and thyroid hormones using the TMI classification. However, in the current study, no correlations between vitamin D concentration and either BMI or TMI were found. Subclinical hypothyroidism is defined as elevated TSH levels with normal total thyroxine (T4) or fT4 (23) concentrations. Subclinical hypothyroidism is known to be common in obesity (23). According to BMI values, all patients with elevated TSH levels were obese. However, all patients with normal TMI had normal TSH values. This may indicate that normal TMI can also mean thyroid gland functions have not yet been impaired. This issue remains to be studied and discussed.

### Study Limitations

The nature of this study required us to rely on data from medical records. Retrospective design and small sample size were the main limitations of our study. In this retrospective study, waist circumference data could not be evaluated from records. In addition, we did not include blood pressure measurements due to the possibility of obtaining unreliable results under suboptimal conditions, where the white coat hypertension effect could not be ruled out (optimal conditions would include seated subjects with a 5-min rest and use of an appropriately-sized cuff). Also, abdominal USG results were limited to patients with IR, due to financial constraints.

## Conclusion

In conclusion, classification by TMI may risk overlooking IR. However, if it is assumed that liver enzymes are elevated as a finding of visceral adiposity, TMI can be used as an auxiliary parameter to show the visceral effects of adiposity. Normal TMI may indicate that visceral organ functions have not deteriorated or visceral organ damage has not yet begun. Thus, TMI can provide different benefits in clinical practice. On the other hand, BMI should be continued to be used because there is a huge body of work on its utility until there are more studies based on TMI. We recommend that BMI and TMI may have different advantages and it would be more appropriate to use them together in clinical practice. Age-specific TMI cut-offs for screening high adiposity; and to compare TMI-based and BMI-based references for indicating visceral adiposity in Turkish children and adolescents are also needed. Overall, there is a need for more and larger prospective studies of the utility of TMI. These would eventually lead to the establishment of both national and international standards for TMI.

## Figures and Tables

**Table 1:**
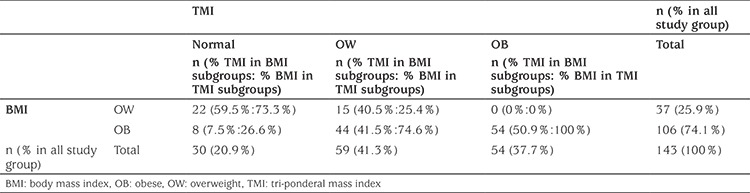
Distribution of groups according to body mass index and tri-ponderal mass index

**Table 2:**
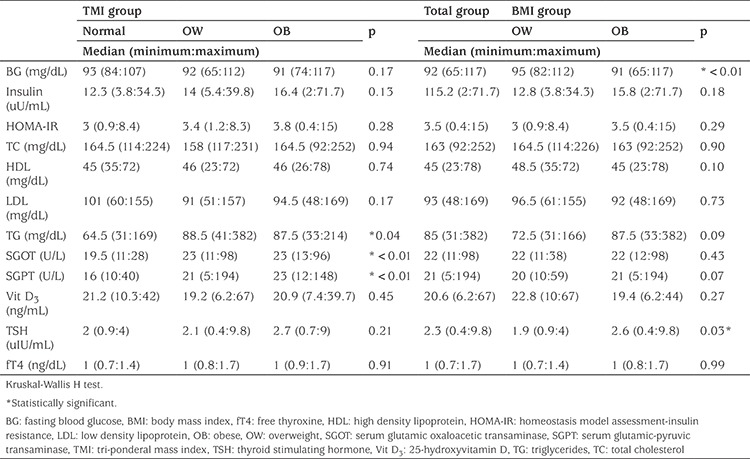
The median, minimum and maximum values of variables in the study sample and in groups classified by tri-ponderal mass index and body mass index-standard deviation score

**Table 3:**
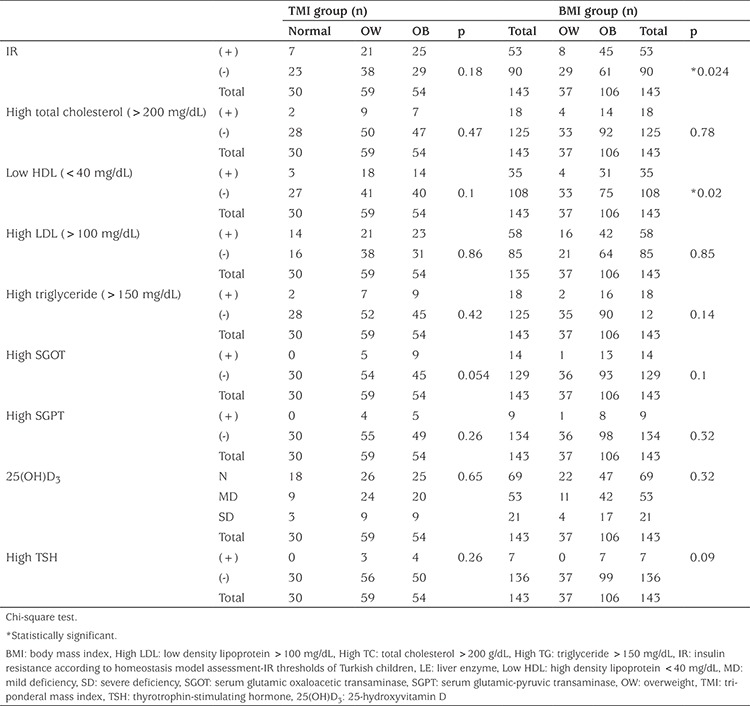
Frequency of pathologic biochemical parameters according to body mass index and body mass index groups

**Table 4:**
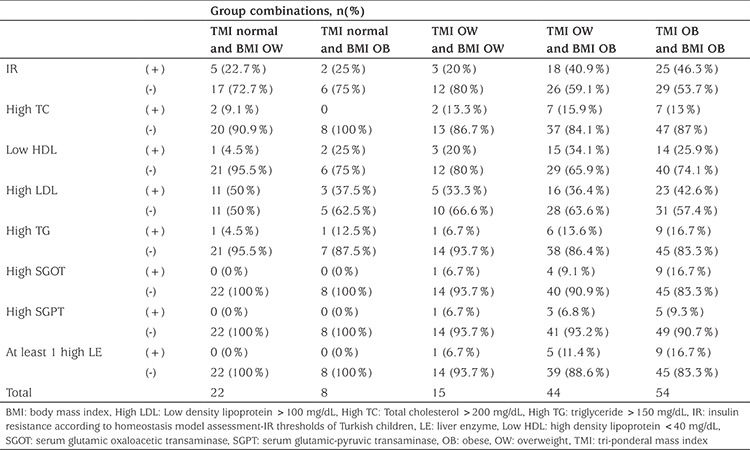
Comparison of variables between groups according to the group combinations of body mass index and tri-ponderal mass index

**Table 5:**
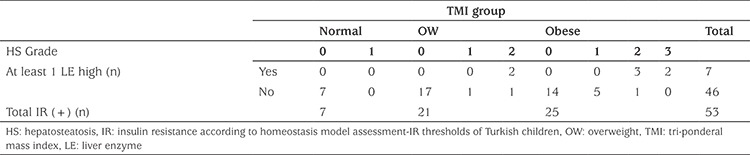
Frequency of high liver enzymes and hepatosteatosis in insulin resistant cases
